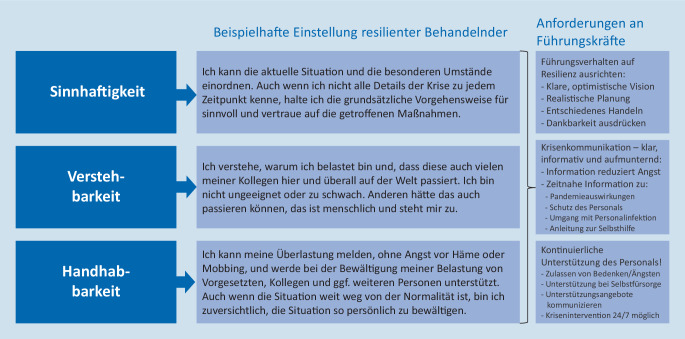# Handlungsempfehlung: Stärkung der Resilienz von Behandelnden und Umgang mit Second Victims im Rahmen der COVID-19-Pandemie zur Sicherung der Leistungsfähigkeit des Gesundheitswesens

**DOI:** 10.1007/s40664-020-00405-7

**Published:** 2020-09-02

**Authors:** R. Strametz, M. Raspe, B. Ettl, W. Huf, A. Pitz

**Affiliations:** 1 Wiesbaden Business School, Hochschule RheinMain, Wiesbaden, Deutschland; 2 Aktionsbündnis Patientensicherheit e. V., Alte Jakobstr. 81, 10179 Berlin, Deutschland; 3 Medizinische Klinik m. S. Infektiologie und Pneumologie, Charité – Universitätsmedizin Berlin, Berlin, Deutschland; 4 Karl Landsteiner Institut für klinisches Risikomanagement, Wien, Österreich; 5 Klinik Hietzing, Wiener Gesundheitsverbund, Wien, Österreich; 6 Professur Sozial- und Medizinrecht, Hochschule Mannheim, Mannheim, Deutschland

**Keywords:** Patientensicherheit, Patientenversorgung, Psychische Belastung, Kommunikation, Führungsverhalten, Patient safety, Patient care, Psychological strain, Communication, Leadership

## Abstract

Der Begriff Second Victim beschreibt eine an der Patientenversorgung beteiligte Person, die durch eine außergewöhnliche Situation in der Patientenversorgung selbst traumatisiert wird. Dieses in der Öffentlichkeit noch weitgehend unbekannte, aber weit verbreitete Phänomen wird durch die COVID-19-Pandemie verschärft und birgt das Risiko, durch eine ausgeprägte psychische Überlastung der Behandelnden Gesundheitssysteme zusätzlich unter Druck zu setzen. Dies stellt sowohl für die Patienten als auch für die Mitarbeitersicherheit eine ernstzunehmende Gefahr dar. Das Second-Victim-Phänomen ist gut erforscht und bedarf einer zweigleisigen Strategie. Einerseits müssen Second Victims in einem flächendeckenden, möglichst niederschwellig erreichbaren gestuften System schnell, persönlich und vertraulich unterstützt werden. Andererseits kommt der Stärkung der Resilienz aller Behandelnden besondere Bedeutung zu. Die Resilienz und damit die langfristige Leistungsfähigkeit der Behandelnden kann durch eine besondere Berücksichtigung im Führungsverhalten und in der Krisenkommunikation nachhaltig unterstützt werden. Sie leistet somit sowohl kurzfristig als auch nachhaltig einen positiven Beitrag für die Patientensicherheit und damit die Überlebenschancen vieler Patienten während und nach der COVID-19-Pandemie.

## Abstract (en)

The term second victim describes a person involved in patient care who, due to an extraordinary patient care situation, also becomes traumatized. This phenomenon is largely unknown to the general public, although it is widespread, and is being exacerbated by the COVID-19 pandemic. Pronounced psychological strain among clinicians entails the risk of increasing pressure on the healthcare system even further. The ensuing threat to the safety of both patients and staff needs to be taken seriously. The second victim phenomenon is extensively researched and requires a two-pronged strategy. Second victims need fast, personal and confidential support within a comprehensive, easily accessible, stratified system and reinforcing clinicians’ resilience is crucial. Leadership and appropriate crisis communication can sustainably support clinicians’ resilience, and thus their ability to function effectively in the long term. Consequently, management can make both a short-term as well as a sustainable contribution to patient safety and therefore increasing the chances of survival for many patients during and after the COVID-19 pandemic.

Die vorliegende Handlungsempfehlung soll Führungskräfte in klinischen und administrativen Bereichen der Gesundheitsversorgung für die Problematik der Traumatisierung von Behandelnden sensibilisieren, die durch die aktuellen psychischen Belastungen im Rahmen der COVID-19-Pandemie ebenso wie die Infektion von Behandelnden selbst weltweit auftreten.

Sie soll durch Optimierung der Führungsinstrumente und der Krisenkommunikation die Resilienz von Behandelnden und Führungskräften stärken und so helfen, das Risiko der Überlastung der Gesundheitsversorgung zu minimieren.

Hierzu wird in einer Kurzdarstellung unter Würdigung verfügbarer Evidenz und anerkannter Empfehlungen, aber auch aktueller Situationsberichte aus Krisengebieten, eine Empfehlung für das praktische Vorgehen vor Ort gegeben.

Dieses Dokument konzentriert sich auf den stationären Bereich der Akutversorgung insbesondere aufgrund der vorhandenen Evidenz. Eine Berücksichtigung entsprechender Strategien zur Stärkung der Resilienz wird jedoch für alle Bereiche der Gesundheitsversorgung dringend angeraten, da die außergewöhnlichen Belastungen sich nicht nur auf die stationäre Akutversorgung beschränken.

Das Dokument gibt den gegenwärtigen Stand des Wissens wieder, der sich im Rahmen der aktuellen dynamischen Entwicklung ändern kann. Hinweise und Ergänzungen an die angegebene Kontaktadresse sind ebenso wie die Verbreitung und/oder Übersetzung des Papers unter Angabe der Quelle jederzeit herzlich willkommen!

Die Autoren

Wiesbaden, Berlin, Wien und Mannheim,

14. April 2020

## Was ist ein Second Victim?

Der Begriff Second Victim wurde im Jahr 2000 von Albert W. Wu für Behandelnde eingeführt, die durch einen selbst begangenen Fehler traumatisiert werden [1]. Der Begriff wurde 2009 von Scott et al. erweitert und beschreibt mittlerweile „eine medizinische Fachperson, die durch einen unvorhergesehenen Zwischenfall am Patienten, einen medizinischen Fehler und/oder einer Verletzung des Patienten selbst zum Opfer wird, da sie durch dieses Ereignis traumatisiert wird“ [2]. In diesem Kontext kann die aktuelle Situation der COVID-19-Pandemie und die damit verbundenen Ausnahmesituationen in den Behandlungseinrichtungen als unvorhergesehener Zwischenfall angesehen werden [3, 4], auch wenn zum jetzigen Zeitpunkt in Deutschland noch keine Triagierungen aufgrund von Überlastungssituationen vorgenommen werden müssen. Diese Einschätzung ist kongruent mit einer Studie von Waterman et al., die zeigen konnte, dass auch Beinahe-Schadenfälle („near misses“) vergleichbare Belastungen bei einem hohen Teil der Befragten ausgelöst haben [5]. Außerordentliche Maßnahmen, steigende Infektionszahlen, insbesondere auch das unter Behandelnden erhöhte Risiko einer Infektion und die in der Zahl zunehmenden schwerwiegenden und letalen Verläufe der Erkrankung, belasten die Behandelnden aller Versorgungsbereiche nicht nur physisch, sondern vor allem auch emotional [6, 7].

## Wie viele Behandelnde sind Second Victims?

Das Second-Victim-Phänomen ist im angloamerikanischen Raum im Bereich der stationären Akutversorgung gut untersucht. Während eine Metaanalyse von Seys et al. aus dem Jahr 2013 Second-Victim-Prävalenzen von 10–42 % aller Befragten angibt [8], gehen aktuellere Studien und eigene in Publikation befindliche Erhebungen aus der Zeit vor der COVID-19-Pandemie von Prävalenzen über 50 % alleine im Rahmen der ärztlichen Weiterbildung aus [9]. Nach Einschätzungen von Experten werden alle Behandelnden früher oder später einmal im Laufe ihres Berufslebens Second Victim [10].

Berichte aus vergangenen Krisensituationen wie der Severe Acute Respiratory Syndrom(SARS)-Pandemie aus dem Jahr 2003 zeigen, dass bis zur Hälfte aller Behandelnden der SARS-Patienten durch das Ereignis akute psychische Reaktionen, Burnout oder eine posttraumatische Belastungsstörung zeigten [6].

Aus aktuellen Berichten italienischer Ärztinnen und Ärzte ist davon auszugehen, dass die in der Literatur beschriebenen Prävalenzen, die sich sonst auf einen Zeitraum mehrerer Jahre oder sogar des gesamten Berufslebens beziehen, binnen weniger Wochen erreicht worden sind und nicht nur die individuelle Gesundheit der Betroffenen, sondern die Leistungsfähigkeit des gesamten Gesundheitssystems massiv beeinträchtigt haben.

*Basierend* *auf Erfahrungen vergangener Krisensituationen (SARS, 9/11 etc.) und den aktuellen Berichten aus dem italienischen Gesundheitssystem, ist davon auszugehen, dass eine systemrelevante Anzahl von Behandelnden durch die aktuelle Situation der COVID-19-Pandemie auch in Deutschland bereits Traumatisierungen als Second Victim erfahren hat oder von dieser Traumatisierung akut bedroht ist.*

## Was sind die Folgen einer Second-Victim-Traumatisierung?

Laut Selbstauskunft betroffener Second Victims verarbeiten bis zu zwei Drittel aller Befragten das zugrundeliegende Ereignis dysfunktional [5, 11–14]. Dies kann sich beispielsweise äußern in:

- Schlafstörungen
- Verlust an den Glauben in eigene Fähigkeiten
- Schuldgefühlen, Isolation, Depression
- Wiedererleben der Situation (Flashbacks)
- Medikamenten- und/oder Alkoholkonsum

In einer Studie unter Anästhesisten von Gazoni et al. gaben 10 % aller betroffenen Second Victims an, sich von diesem Ereignis nie erholt zu haben [15]. Die Folgen für die Betroffenen sind individuell dramatisch und können zur posttraumatischen Belastungsstörung, zur Berufsaufgabe und schlimmstenfalls sogar zum Suizid führen [16]. Neben den Second Victims sind aber auch künftige behandelte Patienten betroffen durch die erhöhte Fehleranfälligkeit der Second Victims aufgrund der verminderten Leistungsfähigkeit und der permanenten Beschäftigung mit dem zurückliegenden Vorfall [17]. Ebenfalls berichtet werden Verhaltensänderungen im Berufsleben bis hin zu defensiver Medizin und absicherndem Verhalten, was im Kontext der COVID-19-Pandemie auch zu klinischen Fehleinschätzungen zu Lasten aller Beteiligten führen kann [18].

*Damit ist nach jetzigem Erkenntnisstand davon auszugehen, dass unzureichend berücksichtigte psychische Belastungen der Behandelnden den Zeitpunkt der Überlastung des Gesundheitssystems zeitlich deutlich vorverlegen bzw. die effektive Gesamtkapazität des Gesundheitssystems deutlich reduzieren.*

## Wie kann Second Victims geholfen werden?

Basierend auf der qualitativen Patientensicherheitsforschung zur Second-Victim-Problematik in den USA wurden in den letzten Jahren in immer mehr Gesundheitseinrichtungen Hilfsprogramme für Second Victims etabliert, wie beispielsweise das RISE-Programm der Johns Hopkins Universität [19], das forYOU-Programm an der Universität Missouri [11] oder die Medically Induced Trauma Support Services (MITSS; [20]). Die Evaluation einzelner Programme zeigt sowohl einen positiven medizinischen Effekt [19] als auch eine Kosteneffektivität [21], wenn die Kosten der Hilfsprogramme mit der Reduktion des finanziellen Schadens von Berufsaufgaben Behandelnder verglichen werden.

Im deutschsprachigen Raum sowie anderen europäischen Ländern [22] gibt es bislang nur vereinzelte ehrenamtliche Initiativen wie den Verein PSUakut e. V. [23] oder das EMPTY-Programm der Young DGINA (Deutsche Gesellschaft für Interdisziplinäre Notaufnahme).

*Flächendeckende und niederschwellig erreichbare Unterstützungsprogramme für Behandelnde existieren im deutschsprachigen Raum derzeit nicht.*

**Figure 1:**
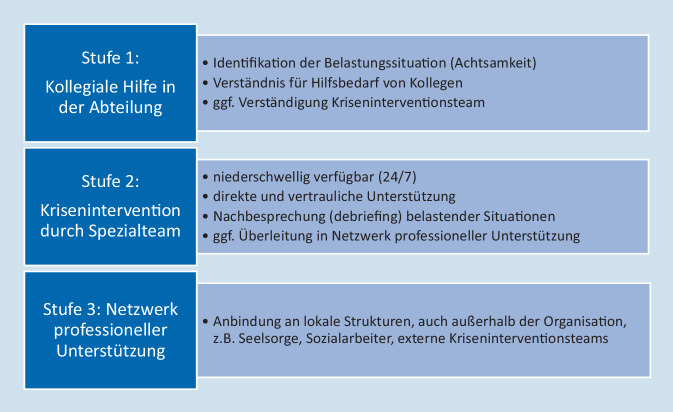

Ziel und gemeinsamer Kern aller bisherigen Hilfsprogramme ist es, durch eine niederschwellig erreichbare, rund um die Uhr verfügbare, gestufte Kriseninterventionsstrategie Second Victims schnell zu unterstützen, um die Erfahrung bestmöglich verarbeiten zu können. Dies alles sollte zudem in einer von Wertschätzung geprägten Atmosphäre stattfinden, in der Belastungen als menschliche Reaktion und nicht als Ausdruck charakterlicher Schwäche verstanden werden. In der Untersuchung des natürlichen Verlaufs konnte gezeigt werden, dass viele Second Victims mit optimaler Unterstützung an dem traumatisierenden Ereignis sogar wachsen können und gestärkt und mit voller Leistungsfähigkeit aus diesem Ereignis hervorgehen. Die Merkmale dieser Unterstützungsangebote basieren auf dem in Abb. 1 dargestellten Modell, das je nach Bedarf um eine Stufe eskaliert werden kann.

Basierend auf allgemeinen Empfehlungen können folgende Maßnahmen vor Ort helfen, Effekte von Second-Victim-Traumatisierungen zu vermindern [11, 14, 24]:

- Kurze Auszeit von der klinischen Tätigkeit anbieten bzw. sicherstellen auch bei Personalknappheit (ein dauerhafter Ausfall wäre die schlechtere Lösung)
- Aktives kollegiales Gesprächsangebot, nicht nur bei vermuteten Fehlern, sondern in regelmäßigen Abständen
- Routinehafte kurze aber effektive Debriefings belastender Situationen/Schichten
- Einfühlsame, aber eindeutige und klare Sprache
- Grundsätzliche Bestätigung der fachlichen Kompetenz und Bestärkung des Selbstwertgefühls des Mitarbeiters
- Emotionen und Ängste zulassen
- Fachliche Unterstützung und Rückversicherung im klinischen Arbeiten anbieten
- Bei Fehlern in der Behandlung Beteiligten eine Rolle bei der Fehleranalyse geben; über Ergebnisse informieren
- Aufmerksame Beobachtung, um Isolierung und Rückzug frühzeitig zu erkennen
- Vermeiden und Ächtung von Lästereien, Mobbing, Schuldzuweisungen und Herabwürdigungen der Beteiligten (um Hilfe zu bitten ist kein Zeichen von Schwäche, sondern menschlich und verantwortungsbewusst gegenüber seinen Patienten)

## Wie können Second-Victim-Traumatisierungen vermieden werden?

Die Agency for Healthcare Research and Quality (AHRQ) benennt in ihrem Framework für Hochzuverlässigkeitsorganisationen Resilienz als einen der fünf kritischen Faktoren [25]. Resilienz, also die Widerstandsfähigkeit von Individuen gegenüber belastenden Situationen, wurde begrifflich von Aaron Antonowsky wesentlich geprägt. Das von ihm als Voraussetzung für Resilienz definierte Kohärenzgefühl besteht aus den drei Komponenten Verstehbarkeit, Sinnhaftigkeit und Handhabbarkeit. Bezogen auf die COVID-19-Pandemie und unter Einbeziehung der aktuellen Empfehlungen von Wu et al. [7] können für Führungskräfte folgende in Abb. 2 dargestellte Empfehlungen abgegeben werden.

**Figure 2:**